# Assessment of Chemical, Mechanical, and Microscopic Properties of Novel Self-Adhesive Dental Composites

**DOI:** 10.1055/s-0045-1813036

**Published:** 2025-12-23

**Authors:** Zainab Sultan, Nayab Amin, Nawshad Muhammad, Heba Mahmoud Ashi, Usama Siddiqui, Muhammad Zeeshan, Saad Liaqat, Zohaib Khurshid

**Affiliations:** 1 Department of Primary and Secondary Healthcare, Lahore, Pakistan; 2 Department of Dental Materials, Institute of Basic Medical Sciences, Khyber Medical University, Peshawar, Pakistan; 3 Department of Dental Materials, Rehman College of Dentistry, Peshawar, Pakistan; 4 Department of Dental Public Health, Faculty of Dentistry, King Abdulaziz University, Jeddah, Saudi Arabia; 5 Department of Dental Materials, Niazi Dental College, Sargodha, Pakistan; 6 Department of Prosthodontics and Dental Implantology, College of Dentistry, King Faisal University, Al-Ahsa, Saudi Arabia; 7 Center of Excellence for Regenerative Dentistry, Department of Anatomy, Faculty of Dentistry, Chulalongkorn University, Bangkok, Thailand

**Keywords:** adhesion, composites, properties

## Abstract

**Objective:**

To synthesize and characterize a novel self-adhesive bioactive and antibacterial dental composite with enhanced mechanical properties.

**Materials and Methods:**

All components were procured from Sigma Aldrich, United States. Commercially available Filtek Z250 and Nexcomp were used as control groups (C1 and C2, respectively). Four novel dental composite groups (C3, C4, C5, and C6) were prepared by mixing monomer solution and inorganic fillers (silica, chlorhexidine, MCPM, and β-TCP **)** in varying concentrations. The resulting dental composites were assessed for chemical, mechanical, and microscopic characterizations. Structural analysis and degree of conversion (DC) of prepared samples were evaluated with Fourier transform infrared spectroscopy (FTIR), while the microscopic interface was evaluated by scanning electron microscope (SEM). Mechanical characterizations included the biaxial flexural strength (BFS) test and the shear bond strength (SBS) test. The obtained data were then analyzed by SPSS version 21.0. Descriptive statistics were used to describe all the values for BFS, SBS, and DC. One-way analysis of variance and post hoc Tukey's test were used to compare the mean values. A *p* -value of less than 0.05 was considered statistically significant.

**Results:**

FTIR results showed a major alteration in DC by calculating the differences in unpolymerized and polymerized aliphatic (1,638 cm ^−1^ ) and aromatic (1,608 cm ^−1^ ) peaks. It was reduced due to the increased quantity of fillers. Commercial composite showed the least DC value, whereas C3 showed higher DC as compared to other novel composites. For BFS, all the groups showed statistically significant differences except C4, whereas SBS results showed an insignificant difference among all the groups. SEM images of the dentin composite interface showed that C1 and C2 composites were not properly bonded to the dentin. The novel dental composite (C3, C4, C5, and C6) showed good bonding with the dentin.

**Conclusion:**

The novel C3 dental composite showed high DC and BFS, whereas C4 had higher SBS but lower than C2. Moreover, effective bonding was achieved with C3 novel composite. It is crucial to optimize the monomer-to-filler ratio for the development of durable bioactive self-adhesive composites with antibacterial property.

## Introduction

Caries is a multifactorial disease that leads to demineralization of tooth structure. 1 It causes localized damage to dental hard tissues due to acidic products produced by fermenting bacteria present in plaque biofilm. It is one of the main causes of oral discomfort, infection, dysfunction of stomatognathic system, and finally tooth loss. 2 Therefore, carious tooth structure needs to be repaired if damage is not progressed to a stage of tooth loss or more serious infection. Caries removal is done by drilling, followed by filling of the lost tooth structure or cavity with direct restorative material. 3

Different restorative materials like amalgam, glass ionomer cements, resin-modified glass ionomer cements, compomers, and composites are used to fill the cavities. 4 Resin-based dental composites (RBCs) have been used for over 50 years as restorative materials due to their superior aesthetics, versatility, and acceptable clinical results. 5 Despite their growing popularity and wide application range, they have concerns such as secondary caries formation at tooth–composite interface leading to material dislodgement and hence failure. 6 These bacteria lead to continuous demineralization of dentin beneath the restoration and consequently the formation of secondary caries. To overcome this problem, the composition of RBCs has been altered with fillers such as antibacterial agents, and bioactive materials that release calcium and phosphate ions to encourage remineralization and antibacterial activity. 7 8

A large variety of calcium phosphates, such as amorphous calcium phosphate (ACP), hydroxyapatite (HA), mono-calcium phosphate (MCPM), di-calcium phosphate (DCP), and tri-calcium phosphate (TCP), are incorporated. In vitro studies have demonstrated that dental composites containing ACP can remineralize tooth lesions by releasing supersaturated ions. Also, TCP and reactive calcium dihydrogen phosphate have been used in combination recently. TCP controls the water absorption of RBCs and the dissolution of calcium dihydrogen phosphate. The bioactivity and antibacterial properties of a light-cured resin containing these calcium phosphates, along with chlorhexidine (CHX), were investigated. High content of calcium phosphates reduced the mechanical properties. 9 Moreover, TCP, when used in various resin-based composites, reacts with water and reprecipitates as anhydrous monetite in an acidic environment, thus promoting remineralization. 10 Although remineralization is achieved by the addition of bioactive materials, the lack of antibacterial properties is also a problem encountered with RBCs. 8 Several antibacterial agents such as strontium fluoride, silver, and chlorhexidine (CHX) are in use to resolve this problem. CHX is effective against many bacterial infections and is widely prescribed in dentistry. 11 It acts by destabilizing the outer membrane of the bacteria. Despite its wide application, CHX has the potential to reduce cell viability through mitochondrial damage. This effect is dependent on both concentration and time. 12 Recently, CHX has been added in minor concentrations in mouthwashes. 13 Thus, dental composites containing CHX have been studied extensively. 11 Greater levels of CHX reduce the degree of polymerization, which leads to increased loss of organic constituents, and increased CHX and MCPM increase CHX diffusion through the matrix phase, and they also increase water sorption. 14 When CHX was used along with calcium phosphate and calcium fluoride nanoparticles, it reduced the formation of biofilm and metabolic activity of bacteria and acid production. 15

As stated earlier, the addition of both antibacterial agents and bioactive agents negatively affected the mechanical properties of RBCs. Therefore, strengthening fillers may be incorporated along with these agents. Over the past 10 years, silica has become more popular as a filler in resinous materials. Adding these fillers to composites has improved their elastic modulus, strength, toughness, stiffness, hardness, and fatigue resistance. The optimal concentration varies in the literature and depends on the selected polymerizable resin. However, a higher proportion of silica adversely affects the resin's viscosity and restricts its use. 16

Given the description above, the present study aimed to synthesize and characterize a novel self-adhesive composite that possesses improved mechanical properties in addition to bioactivity and antibacterial properties.

## Materials and Methods

In this study, all chemicals, including monomers and photoinitiators to prepare the novel resin-based composites, were of analytical grade and purchased from Sigma Aldrich, the United States. Commercially available Nexcomp (META BIOMED) and Filtek Z250 (Universal Restorative 3M ESPE, Germany) were used as control groups (C1 and C2, respectively). Their compositions are given in Table 1 . The novel material was based on dental resin monomers triethylene glycol dimethacrylate (TEGDMA), urethane dimethacrylate (UDMA), and hydroxyethyl methacrylate (HEMA). The initiator and activator used in this study were camphorquinone (CQ) and *N, N* -dimethyl- *p* -toluidine (DMPT). The salinized silica powder was received from the Interdisciplinary Research Centre in Biomedical Materials (IRCBM), COMSATS University, Islamabad, Lahore Campus, Pakistan.

**Table 1:** Various commercial groups along with composition

Symbol	Commercial dental composite	Monomers wt.%	Fillers wt.%
C _1_	(Nexcomp META BIOMED)	25	75
C _2_	Commercial dental composite (Filtek Z250)	40	60

### Novel Dental Composite Preparation

#### Preparation of Monomer

The monomer was prepared in a controlled environment. A glass beaker was wrapped with foil to avoid contact with light. The ingredients were mixed according to the given weight percentage ratio: UDMA (68 wt.%), TEGDMA (25 wt.%), HEMA (5 wt.%), CQ (1 wt.%), and DMPT (1 wt.%). Small amounts of ingredients were weighed first, and large amounts of ingredients were weighed last. Viscous monomer like UDMA was added last. Mixing of all components was done for 10 minutes with the help of a magnetic stirrer at 300 rpm before adding UDMA. The mixture was mixed further for 15 minutes after the addition of UDMA.

#### Paste Mixing

To prepare novel dental composites, the monomer solution prepared earlier was stirred at ambient room temperature (25 °C) to get a homogenous solution or to avoid any sedimentation. Then, the silica (30–50%) powder, CHX (1–10%), MCPM (1–10%), and β-TCP (1–10%), which were optimized initially to set the ratio, were added manually to the monomer solution to achieve the desired consistency. These reinforcing materials were added incrementally, and after complete addition, the mixture was stirred at 450 rpm at ambient room temperature for an hour. Ten mL of 99.5% ethanol was added to the solution to help mixing. Aluminum foil was used to cover the beaker, and holes were made to facilitate ethanol evaporation. After overnight stirring, the obtained composites were packed in air-tight and dark vials for further use. The composition and ratio of materials used in the study are described in Table 2 .

**Table 2:** Various experimental groups along with composition

Symbols	Experimental groups	Monomers wt.%	Fillers wt.%		
			CHX	SiO _2_	MCPM and β-TCP
C3	Novel dental composites	60	0	40	0
C4	Novel dental composites	55	5	40	0
C5	Novel dental composites	45	5	40	10
C6	Novel dental composites	50	0	40	10

### Sample Preparation and Characterizations

The samples were prepared according to ISO standards and manufacturer's guidelines. The following characterizations were performed.

#### Fourier Transform Infrared Spectroscopy

Four discs of each formulation were used, and Fourier transform infrared spectroscopy (FTIR) was performed before and after curing of the sample to estimate the degree of conversion (DC). FTIR Thermo Nicolet 6700 (United States) with a photoacoustic cell was used as a detector. The Teflon mold, having a thickness of 1 mm and a diameter of 10 mm, was placed onto the FTIR diamond. The molds were filled with the dental composite, and the filled molds were instantly covered with an acetate sheet and glass slab to expel the excess material. Spectra were collected over the region of 4,000 to 400 cm ^−1^ at resolution of 8 cm ^−1^ and averaging 256 scans. Then the dental composite was cured for 40 seconds using Woodpecker 470 nm LED light. And then spectra were collected. The data was analyzed by using OMNIC software. Monomer conversion and polymerization shrinkage were calculated as follows:



where DC denotes the degree of monomer conversion and R is the ratio of peak heights at 1,638 and 1,608 cm ^−1^ of polymerized and unpolymerized disc sample.

#### Biaxial Flexural Strength Testing

Teflon molds having 2-mm thickness and 10-mm diameter were positioned onto an acetate sheet on a glass slab. The molds were filled with the dental composite, and the filled molds were instantly covered with another acetate sheet and a glass slab to expel the excess material. The dental composite was cured for 40 seconds using Woodpecker 470 nm LED light. The acetate sheet and glass slab were detached after curing, followed by the smoothing of discs using a sharp razor blade. Each disc was stored in 10 mL of distilled water in a glass beaker. The glass beakers with composite discs were stored for 24 hours at 37 °C.

ISO 4049 was used to measure biaxial flexural strength (BFS). Ten discs of each composition were used to measure BFS. Hydrated sample of disc was positioned on a ring support with a radius of 4 mm, and then the discs were loaded by a spherical tip in the M500-50AT Universal Testing Machine. BFS was calculated using the ball-on-a-ring method.

BFS was calculated by means of the following formula.



where *σ* is the BFS in MPa, Lmax is the maximum load in *N* , *a* is the support radius in mm, and *t* is the average thickness of the specimen in mm.

#### Shear Bond Strength Test

Freshly extracted bovine teeth (cow) were used for sample preparation. They were washed thoroughly under running tap water to get rid of blood and adherent tissue. After thorough cleansing, the teeth were stored in water at 37 °C. Bovine dentin was exposed and following the treatment, both commercial and novel dental composite pastes were applied on the exposed bovine dentin. Acid etching was performed for 20 seconds, followed by thorough water rinsing. After air drying, a dental adhesive was applied and light-cured. To check the adhesion, the composite was dispensed in increments into the plastic tube having a 3 mm diameter and a length of 6 mm. Plastic tubes with a composite were placed on the dentin surface. The testing was completed according to ISO 29022:2013. Shear bond strength (SBS) was determined using a M500-50AT universal testing machine with a “Flat-edge shear fixture” jig. The jig consisted of a metal holder with an adjustable screw to secure the specimen and an adjustable blade, which was used to shear the tube from the dentin. A 50 KN load cell at cross-head speed of 1 mm/min was used. The load at break was recorded, and SBS ( *τ* ) was calculated using equation 2.3, in which *F* is the load at break, and *A* is the bonded area of the cylinder.



#### Scanning Electron Microscopy

Human teeth were used for sample preparation, which were obtained from the Exodontia Department of Punjab Dental Hospital, Lahore, after the informed consent of the patient. Cavities of 3 mm in diameter and 5 mm in depth were created in human dentin to examine the interface and micro-gap formation due to the shrinkage of the composite. Cavities were washed after drilling, and no acid etch or dental adhesive treatment was done.

Then the cavities were restored by placing commercial and novel composites. Each composite was placed in a cavity in 2-mm increments, followed by light curing for 40 seconds. The restored teeth were sectioned vertically using a fast dental handpiece under continuous flow of water to observe the adhesive interface. The surface of dentin was polished, and sputter-coated with gold before the microscopic imaging of interfaces.

The samples were examined by using Scanning Electron Microscope TESCAN VEGA-3 LMU, Czech Republic. Images were captured in the magnification ranging from 1,500× to 5,000× at an accelerated voltage of 10 to 20 kV. Energy-dispersive X-ray EDS analysis was also conducted using Oxford instruments.

## Results

### FTIR

Fig. 1 (A–F) shows a representative spectrum of commercial and novel dental composite materials showing characteristic peaks of dimethacrylate resins. The change in peak heights/intensities before and after curing is shown in Table 3 .

**Table 3:** Change in peak heights/intensities before and after curing with DC

Groups	Peaks (cm ^−1^ )				DC
	1,638 ( *ali* C = C)		1,608 ( *aro* C = C)		
	Absorbance before curing	Absorbance before curing	Absorbance before curing	Absorbance before curing	
**C1**	0.043	0.005	0.031	0.005	40%
**C2**	0.042	0.011	0.044	0.017	52%
**C3**	0.097	0.022	0.035	0.006	77%
**C4**	0.120	0.080	0.060	0.040	65%
**C5**	0.068	0.115	0.033	0.052	43%
**C6**	0.097	0.002	0.041	0.004	56%

**Fig. 1:**
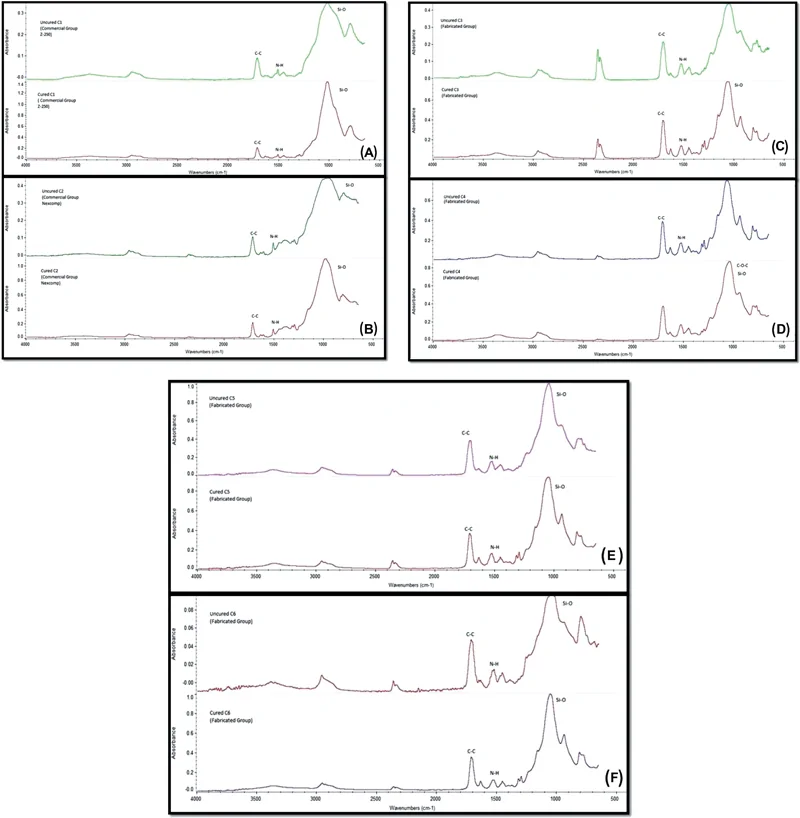
( **A–F** ) Spectrum of composite materials showing characteristic peaks of dimethacrylate resins.

A statistically significant difference ( *p* ≤ 0.05) was detected between DC of composites, where C3 showed the highest conversion rate after polymerization in the following sequence: C3 (77%) > C4 (65%) > C6 (56%) > C2 (52%) > C5 (43%) > C1 (40%).

### Biaxial Flexural Strength

The results of BFS showed that the strength values for C3 were highest, followed by C2, C1, C4, C5, and C6. The corresponding details and standard deviations are given in Fig. 2 .

**Fig. 2:**
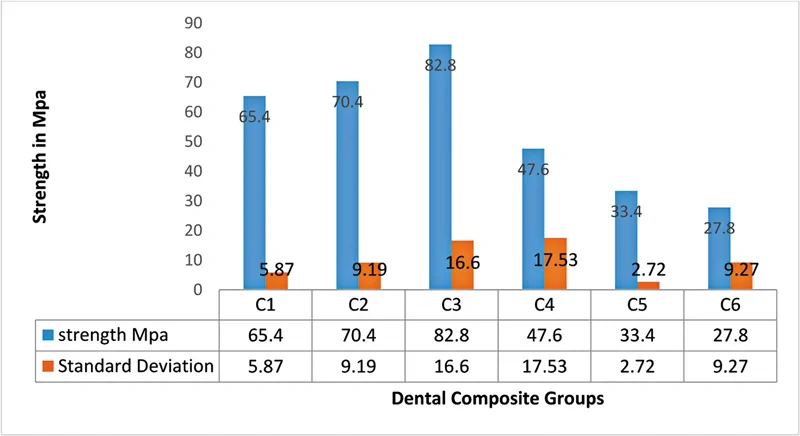
Mean biaxial flexural strength values of control and experimental groups along with standard deviation.

### Shear Bond Strength

The results of SBS showed that the strength values for C2 were highest, followed by C4, C5, C3, C1, and C6. The corresponding details and standard deviations are given in Fig. 3 .

**Fig. 3:**
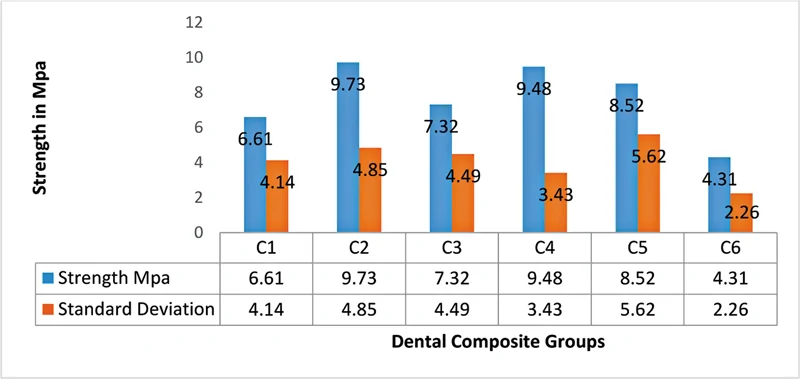
Mean shear bond strength values of control and experimental groups, along with standard deviation.

### Scanning Electron Microscopy

The scanning electron microscope (SEM) images of the interface between various groups are shown in Fig. 4 (1a, 1b to 6a, 6b). It is clearly shown (1a, 1b and 2a, 2b) that C1 and C2, respectively, did not adhere properly to the underlying dentin, while the experimental groups (C3, C4, C5, and C6) adhered properly to the underlying dentin (3a, 3b; 4a, 4b; 5a, 5b; and 6a, 6b, respectively).

**Fig. 4:**
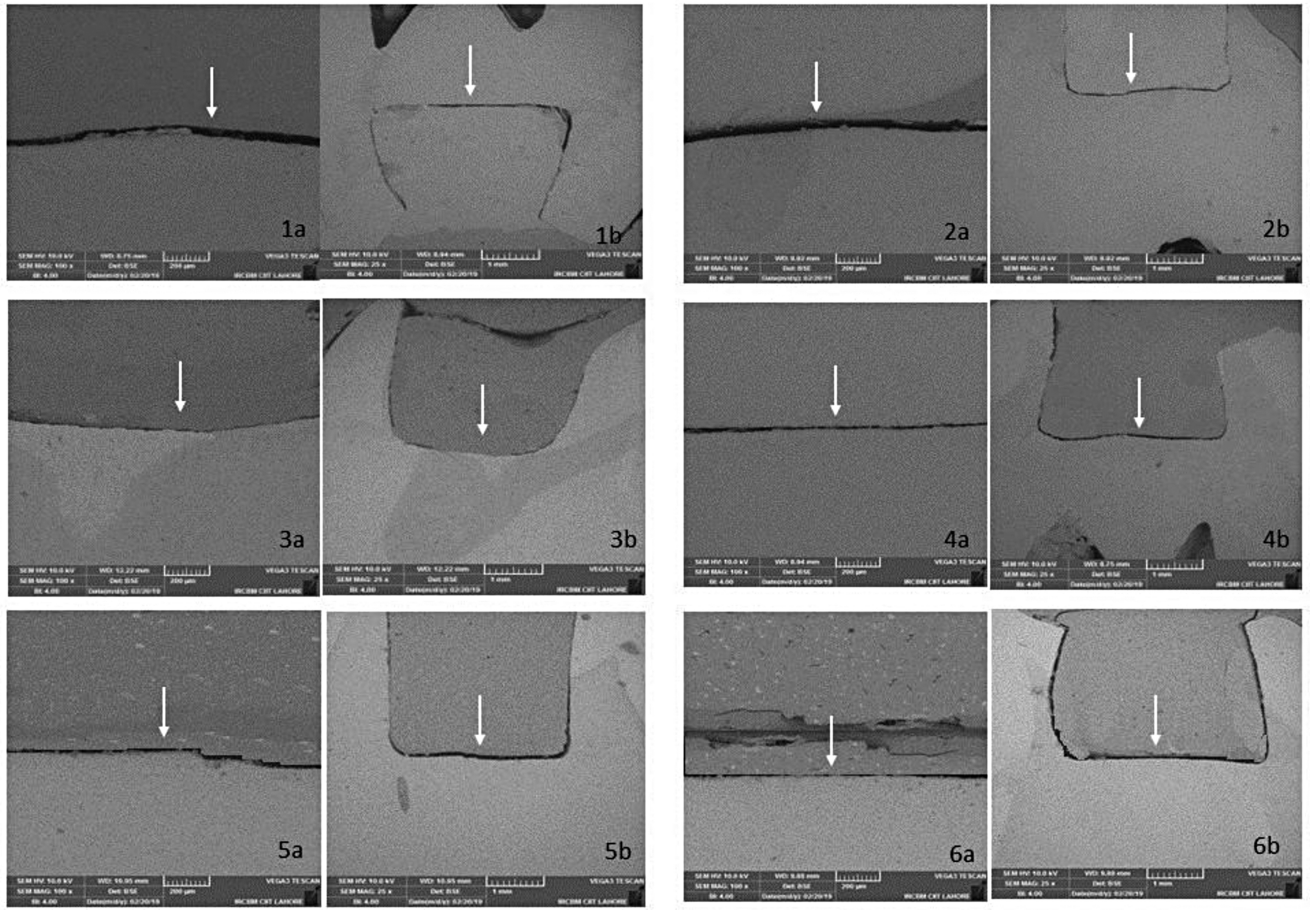
SEM images of control and experimental composites: (1a, b) C1, (2a, b) C2, (3a, b), C3, (4a, b) C4, (5a, b) C5, (6a, b) C6. White arrows in each image indicate the adhesive interface between the composites and the tooth.

## Discussion

The DC of RBCs is evaluated with FTIR. The performance of RBCs is dependent on DC. 17 All mechanical, structural, and organic properties of materials normally increase as the DC of the monomer increases. When the mechanical and structural properties of materials are greater, there is a lower likelihood of restoration, thus reducing the danger of recurrent caries and bacteriological microfiltration. A higher degree of DC significantly lessens the likelihood of cytotoxicity caused by the release of unreacted monomers, which can lead to postoperative sensitivity. Furthermore, polymerization of the monomers could influence the release of reactive substances. The DC of C3 was highest as it contained silica particles. This is due to the fact that silica particles are transparent and allow the passage of light through them, which will subsequently increase DC. The results of composites containing silica in this study are better compared to the previous study in which silica was used as filler. 18 The group C4, which contains CHX and silica, had the results comparable to another study done by Larissa et al in which silica and CHX were used as fillers. 19 The result of the group C6 (silica, MCPM, and β-TCP) showed lower DC as compared to a previous study done by Kangwankai et al. This may be because, in that study, lower percentages of MCPM and β-TCP were incorporated, while more silica was used. In the same study, when DC of Z250 was assessed, the results were almost same as in this study. 20

Mechanical properties of the dental composites should be satisfactory to resist the strong masticatory forces. When in use, the composite materials are exposed to all three types of stresses: tensile, compressive, and shear. The combined form of all three stresses is flexural stress. So, the appropriate method to evaluate mechanical strength is to measure flexural strength.

In this study, the novel composites C3 presented with highest values of BFS, followed by C1 and C2. Greater strength of groups C1 and C2 may be due to high filler loading and larger particle size. These two factors enhance the value of strength in composites. Filler type and shape also affect the strength properties of dental composites. Particles in C2 are spherical, which tends to increase the packing and the strength as well. Besides this, the monomer conversion of C2 was low and the mechanical properties were good. The reason behind this may be due to *bis* -GMA, which is the key ingredient in C2. As, *bis* -GMA is more rigid and less flexible when compared to UDMA. 21

The higher biaxial strength of novel group C3 composite may be due to increased monomer conversion, which results in increased conversion of monomers to polymers and strong cross-linking of polymer. The cross-linking makes the polymer stronger and stiffer. 22 BFS values were decreased by the incorporation of reactive fillers. These fillers impart new properties to the novel dental composite. Reactive fillers absorb water and have the potential to be released from the material. Addition of MCPM and β-TCP reduced the BFS values, which may be due to the reason that it encourages water sorption and consequently, polymer chains will expand due to the absorbed water, and reduce the BFS values of the dental composites. The results of MCPM and β-TCP containing dental composites were almost similar to another study conducted by Regnault et al, in which a mixture of zirconia and calcium phosphate was added as filler in UDMA-based dental composites. 23

When 5% CHX was added, a decline in BFS was seen. This may be since CHX has high solubility in distilled water and high-water sorption, so here also polymer chains will expand due to the absorbed water and will eventually decrease strength. CHX also decreases the wetting ability of monomer, which results in a decline in BFS values, as poor wetting causes increased water bubble incorporation during the mixing of the composite. Additionally, water sorption causes leaching of reactive fillers such as MCPM, β-TCP, and CHX decreasing strength. The results obtained are better than a previous study conducted by Muller et al, in which experimental composites containing silica and CHX were evaluated. 19 This discrepancy may be attributed to the fact that, in their study, silica was pre-loaded with CHX, while in our study they were mixed separately. It was noted that the SBS and BFS values of various groups did not follow the same trend. This may be due to fact that these tests evaluate different properties. SBS reflects the adhesive interface with the tooth structure, whereas BFS measures the bulk strength of the composite. 24 Variations arise due to resin chemistry, filler content, resin chemistry, and polymerization shrinkage. All of these factors affect interface bonding and bulk integrity differently. Additionally, SBS is sensitive to interfacial flaws and testing setup, whereas BFS depends on internal crack resistance, so their trends do not necessarily correlate. 25

RBCs have a greater chance of failure due to secondary caries under restoration. This is because polymerization shrinkage in them leads to micro gaps between the tooth/restoration interface, thus causing micro-leakage and allowing microorganisms to enter the space. 26 In this study, the acidic monomer HEMA was added to provide self-adhesive properties to novel composites that can adhere to dentin without the use of any etching and bonding. Use of HEMA in dental adhesives is known, so it was incorporated into the monomer phase of the novel composites, which improved the bond under various dentin pretreatments. 27 Materials containing HEMA bonded with dentin more conveniently when compared to commercial composites. Etching with phosphoric acid has been in practice for years. 28 To assess adhesion of composites to tooth structure, SBS test was used. This test commonly uses bovine teeth as a substitute for human teeth due to their easier availability, larger surface area, and ethical considerations. 29 Moreover, their composition, bonding behavior, and hardness are identical to human teeth. 30 HEMA improves the bonding of RBCs with dentine; however, by adding reactive fillers such as calcium phosphate and CHX, the shear bond strength was decreased. This may be due to the incorporation of air in the mixture, as these fillers were difficult to mix. The results of group C6 are similar to another study in which MCPM and nisin are added to dental composites containing HEMA. 31 The results of C1 are less compared to the study done by Lee and Park and Syed et al, though both of them used human teeth as compared to bovine to assess the SBS. 32 33

The success of dental restorations in the oral cavity depends on the type of interface between dentin and composites. This study was conducted to calculate several features that may possibly disturb the bond strength, and to see the result of these variables on the interface between dentin and dental composite under SEM. For this purpose, human teeth were preferred as they offer significant clinical information about interfacial and morphological attributes of restorative techniques. Besides this, visualization of human teeth to check for interface under SEM is more accurate compared to bovine teeth. 34 The commercial dental composites could not adhere properly to the underlying dentin. The novel dental composite showed good bonding with the dentin. This showed the experimental composite containing a high monomer percentage has improved adhesive properties due to HEMA incorporation. The results of this study are in accordance with another study done by Van Landuyt et al, which demonstrated that addition of HEMA improves dentin–adhesive interface. 35

## Conclusion

The high demand for restorative materials that combine mechanical strength, bioactivity, and antibacterial properties has led to the investigation of novel self-adhesive composites as possible alternatives to traditional dental composites. The novel C3 composite (60% monomer, 40% silica) demonstrated a high DC and BFS, whereas C4 (55% monomer, 5% chlorhexidine, 40% silica) had higher SBS but lower than C2 (control group composite: Filtek Z250). Moreover, effective bonding was achieved with the C3 novel composite due to an increase in monomer content as compared to other novel composites and also the control group composites. These findings highlight the importance of optimizing the monomer-to-filler ratio to develop next-generation composites having strong mechanical properties with efficient bonding ability and bioactivity.

## Limitations

It is important to recognize the limitations of this research. Both human and bovine teeth were used for different testing, which may give variable results; however, bovine teeth were used as substitutes due to their easy availability and similar structure. The experimental settings did not include thermocycling and water-sorption analysis, which are crucial to simulate oral conditions. Moreover, future research should include biocompatibility tests, bioactivity analysis, antibacterial efficacy, and in vivo assessments to completely verify these novel self-adhesive composites. Additionally, the most effective combination of mechanical strength, antibacterial effect, and bioactivity can only be achieved by optimizing the monomer-to-filler ratio.
